# Positional scanning of natural product hispidol’s ring-B: discovery of highly selective human monoamine oxidase-B inhibitor analogues downregulating neuroinflammation for management of neurodegenerative diseases

**DOI:** 10.1080/14756366.2022.2036737

**Published:** 2022-02-23

**Authors:** Ahmed H. E. Hassan, Hyeon Jeong Kim, Min Sung Gee, Jong-Hyun Park, Hye Rim Jeon, Cheol Jung Lee, Yeonwoo Choi, Suyeon Moon, Danbi Lee, Jong Kil Lee, Ki Duk Park, Yong Sup Lee

**Affiliations:** a Department of Medicinal Chemistry, Faculty of Pharmacy, Mansoura University, Mansoura, Egypt; b Medicinal Chemistry Laboratory, Department of Pharmacy, College of Pharmacy, Kyung Hee University, Seoul, Republic of Korea; c Convergence Research Center for Diagnosis, Treatment and Care System of Dementia, Korea Institute of Science and Technology (KIST), Seoul, Republic of Korea; d Department of Fundamental Pharmaceutical Science, Graduate School, Kyung Hee University, Seoul, Republic of Korea; e Department of Life and Nanopharmaceutical Sciences, Kyung Hee University, Seoul, Republic of Korea; f KHU-KIST Department of Converging Science and Technology, Kyung Hee University, Seoul, Republic of Korea

**Keywords:** Natural products analogues, MAO-B inhibitors, neuroinflammation, neurodegenerative diseases, Parkinson’s disease

## Abstract

Multifunctional molecules might offer better treatment of complex multifactorial neurological diseases. Monoaminergic pathways dysregulation and neuroinflammation are common convergence points in diverse neurodegenerative and neuropsychiatric disorders. Aiming to target these diseases, polypharmacological agents modulating both monoaminergic pathways and neuroinflammatory were addressed. A library of analogues of the natural product hispidol was prepared and evaluated for inhibition of monoamine oxidases (MAOs) isoforms. Several molecules emerged as selective potential MAO B inhibitors. The most promising compounds were further evaluated *in vitro* for their impact on microglia viability, induced production of proinflammatory mediators and MAO-B inhibition mechanism. Amongst tested compounds, **1p** was a safe potent competitive reversible MAO-B inhibitor and inhibitor of microglial production of neuroinflammatory mediators; NO and PGE_2_. *In-silico* study provided insights into molecular basis of the observed selective MAO B inhibition. This study presents compound **1p** as a promising lead compound for management of neurodegenerative disease.

## Graphical Abstract

**Figure:**
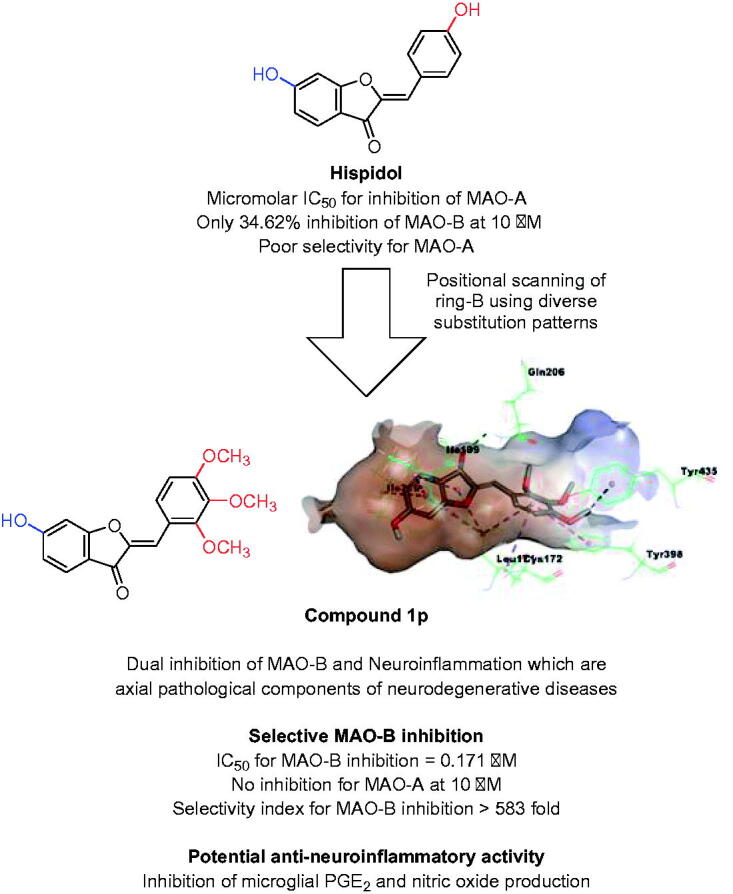

## 1. Introduction

Healthy adult brain is estimated to have 100 billion neurons forming 100 trillion neuronal connections (synapses)^1^. Signals flow through these massive brain’s neuronal circuits require chemical neurotransmitters which affect emotions, thoughts, movements, and memories. Disruption of such intricate brain networking results in divergent neurological disorders including neurodegenerative and neuropsychiatric diseases which afflicts millions of peoples^2^. Unfortunately, the currently available therapeutics are still far from realising complete cure of these neurological diseases and, in addition, such diseases impose a high socioeconomic burden^3^^,^^4^.

Despite the complex etiopathologies of neurodegenerative and neuropsychiatric diseases, common converging mechanisms and manifestations were found shared by these divergent diseases pathways^5–12^. Amongst known aetiological factors, monoaminergic pathway abnormalities were demonstrated as a major contributor and/or manifestation of numerous neurodegenerative and neuropsychiatric diseases^10–12^. Synaptic transmission in monoaminergic system involves dopamine (DA), serotonin (5-HT), noradrenaline (NA), and histamine as chemical neurotransmitters which are catabolised by monoamine oxidases (MAOs). MAOs are mitochondrial membrane-bound enzymes that convert monoamines into the corresponding aldehydes producing hydrogen peroxide, ammonia products of the same reaction. Two isoforms of MAO have been identified; MAO-A which is mainly associated with catabolism of catecholaminergic and serotonergic neurotransmitters; and MAO-B which is mainly associated with catabolism of dopaminergic and histaminergic neurotransmitters. Impairment of monoaminergic signalling *via* upregulation of MAO activity results not only in lowering neurotransmitters’ levels, but also the products of the enzymatic reaction involving reactive oxygen species (ROS) induce oxidative stress; a well-known cause of degenerative neuronal cells’ death^13^^,^^14^. Consequently, monoamine oxidase inhibitors (MAOIs) would enhance the monoaminergic neurotransmission within the brain’s circuits and also protect neuronal cells against cytotoxicity associated with increased MAO activity/expression. Because of limitations of irreversible inhibitors of MAOs including hypertension crisis arising from irreversible inhibition of MAO-A coupled with dietary amines’ intake, development of selective MAO-B inhibitors was addressed^15^. Nevertheless, long-term treatment of neurodegenerative diseases with irreversible MAO-B inhibitors fails due to compensatory mechanisms^16^. In contrast, selective reversible inhibitors have proven to elicit a better therapeutic profile. Successfully, moclobemide; a reversible MAO-A inhibitor, has been approved for treatment of neuropsychiatric disorders, such as depression, whereas safinamide, a reversible MAO-B inhibitor, has been approved for neurodegenerative disorders, such as Parkinson’s disease (PD) and Alzheimer’s disease (AD).

Neuroinflammation is another commonality shared in neurodegenerative and neuropsychiatric diseases. CNS neuroinflammatory response is mainly triggered by microglia releasing inflammatory mediators, such as nitric oxide (NO), PGE_2_, and cytokines as defence mechanisms. However, abnormal activation results in neurotoxic effects and eventually neuronal cells death^17^. Therefore, neuroinflammation is a component in several neurodegenerative diseases including PD, AD, amyotrophic lateral sclerosis (ALS), and frontotemporal dementia (FTD)^17^. This is further supported by multiple evidences demonstrated targeting the neuroinflammatory component in neurodegenerative diseases as a valid treatment approach^18–20^. In addition, neuroinflammation was found as a key contributor to the development and progression of several neuropsychiatric diseases including schizophrenia, depression, and bipolar disorders^21^^,^^22^. A microglia hypothesis for neuropsychiatric disorders was shaped in recognition of their central roles^23^. Eventually, recent literature reports attempted targeting neuroinflammation towards treatment of neuropsychiatric diseases^24^^,^^25^.

Inflammation is an important component of the vicious cycle that exists in several complex diseases such as neurological disorders and cancers^26^^,^^27^. For example, neurodegeneration triggers neuroinflammation which in turn results in the formation of neurotoxic substances that trigger more neurodegeneration. Therefore, the efficacy of a single targeting therapy might be limited for treatment. A polypharmacological molecule impacting multiple pathways involved in such complex diseases might be more effective strategy to break this vicious cycle and achieve better therapeutic effects^28–33^.

Because of the high cost of new drugs development, repositioning or repurposing of previously developed or failed molecules has emerged as a promising strategy in drug discovery and development^2^^,^^34^. In fact, literature reports several successful discoveries after repurposing strategy^35–38^. Herein, we report our efforts to develop multifunctional molecules inhibiting both of human MAO and neuroinflammation based on rational repurposing approach.

## 2. Results and discussion

### 2.1. Design of focussed hispidol analogues library

**Figure 1.**
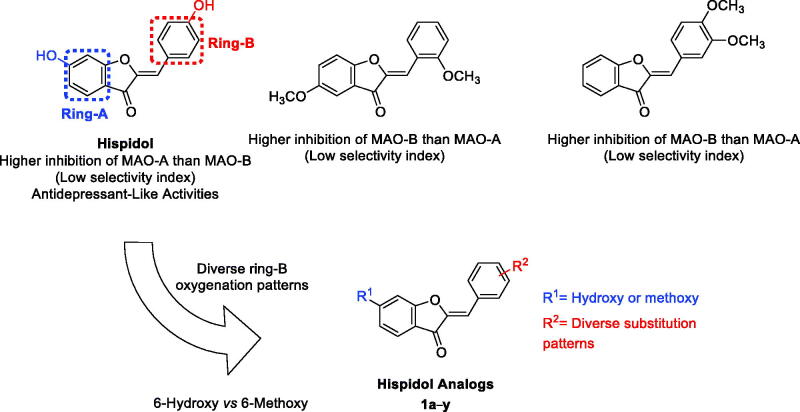
Positional scanning of hispidol’s ring-B towards generation of hispidol analogues’ focussed library based on monoamine oxidases inhibition and selectivity of hispidol and other methoxylated-aurones.

Considering that drug discovery of naturally inspired compounds might be associated with higher success rates including MAOIs^39–42^, hispidol (Figure 1); a 6-hydroxyaurone-based natural product, was chosen as a starting lead compound. It was recently reported to inhibit *in vitro* MAO-A and MAO-B with more selectivity towards MAO-A inhibition as well as to elicit *in vivo* antidepressant-like effects^43^^,^^44^. Previously, aurone was showed as possible scaffold for development of MAOIs^45^. Considering that aurones, as well as other flavonoids, exist in natural with different oxygenation patterns of rings A and B, literature was searched for MAO inhibition by natural/synthetic oxygenated aurones. In contrast to hispidol, the search outcome showed that reported unsubstituted-ring A, 5-hydroxy/methoxy or 4-hydroxy derivatives of aurones with ring B bearing simple hydroxylation/methoxylation patterns elicit almost low selectivity for inhibition of MAO-B (Figure 1)^46^^,^^47^. Based on the reported activity of hispidol, the effort reported herein aims to conduct positional analogue scanning^48^ of hispidol analogues employing hydroxylation/methoxylation patterns of ring B towards identification of molecules with interesting MAO inhibitory profiles and activities. In addition, a recent report has shown some synthetic hispidol analogues to elicit anti-inflammatory effects in *in vitro* LPS-induced macrophages’ model^49^. These points might provide a rationale for hispidol analogues repurposing for development of multifunctional agents modulating monoaminergic signalling and neuroinflammation; both are axial components in the neurodegenerative and neuropsychiatric diseases. Towards this goal, hispidol was selected as a starting point to establish a focussed library of analogues. While the hydroxy moiety is a polar moiety that can act as hydrogen bond donor or acceptor, the methoxy moiety is less polar as well as can act only as hydrogen bond donor. In addition, both hydroxy and methoxy moieties are ubiquitous in natural products. Therefore, and in attempt to gain insights of structure–activity relationship and, as illustrated in Figure 1, the characteristic C-6 hydroxy substitution pattern of ring A found in hispidol was maintained or converted to the natural products ubiquitous methoxy moiety in the designed library. While hispidol possesses one hydroxy moiety at ring B located at C-4′ of the aurone scaffold, this hydroxy was translocated to C-2′ and C-3′ position to explore activity of regioisomers. In addition, members having diversely dihydroxylated ring B were enumerated in the designed library to pattern explore the impact of different dihydroxylation pattern on the activity. Furthermore, inclusion of trihydroxylated ring B among explored patterns was planned. Moreover, exploration of monomethoxylated ring B regiosomers, diverse dimethoxylation patterns of ring B, and trimethoxylated ring B as well as the protruding 4′-methoxymethoxy moiety were considered in the designed library to investigate the impact of these structural modifications on the activity.

### 2.2. Synthesis of targeted library members

**Scheme 1.**
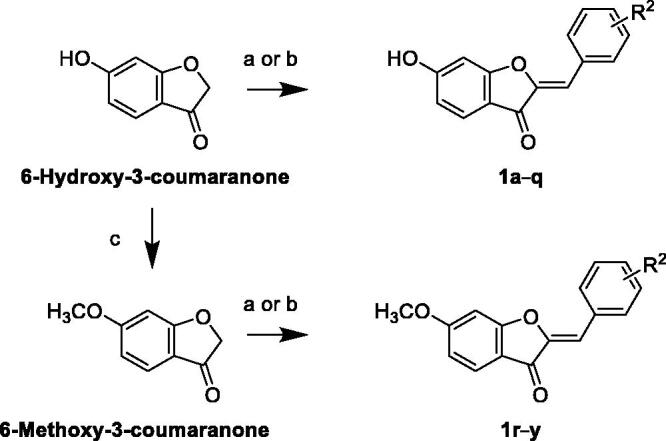
Reagents and reaction conditions: (a) 12 N HCl, ethanol, 60–70 °C, for the specified time; (b) 50% KOH, methanol, 60 °C, or the specified time; (c) methyl iodide, K_2_CO_3_, DMF, and rt, overnight.

Economies of the synthesis including step and atom economy are important consideration in achieving a concise practical synthesis^50–52^. In lieu of this, hispidol analogues **1a–q** possessing C-6 hydroxy feature were synthesised in single step *via* acid-catalysed or base-catalysed cross-aldol condensation of commercially available or 6-hydroxy-3-coumaranone with diversely substituted aldehydes similar to reported procedure to afford the desired compounds (Scheme 1)^49^. To access hispidol analogues **1r–y** possessing C-6 methoxy feature, 6-hydroxy-3-coumaranone was methylated using methyl iodide in presence of potassium carbonate as a base and dimethylformamide as a solvent to yield 6-methoxy-3-coumaranone. Similar to the previously reported procedure, acid-catalysed or base-catalysed cross-aldol condensation of 6-methoxy-3-coumaranone with diversely substituted aldehydes yielded the desired C-6 methoxy members **1r–y** of the desired hispidol analogues library^49^. The structures of unreported compounds were elucidated by ^1^H NMR, ^13 ^C NMR, and HRMS analyses. For known compounds **1a**, **1b**, **1d**, **1e**, **1f**, **1h–1t**, and **1v–1y**, spectroscopic data were in agreement with literature^43^^,^^49^^,^^53–58^.

### 2.3. Biological evaluation

#### 2.3.1. Evaluation of inhibition of different monoamine oxidase isoforms

Two different MAO isoforms are known. Each isoform is correlated with different neurological disorders. Therefore, the designed focussed library of hispidol analogues were evaluated for inhibitory activities against both recombinant human MAO-A and MAO-B isoforms employing previously reported spectrophotometric method^59^. The screening results are summarised in Table 1 and discussed in the following sections.

**Table 1.** Results of *h*MAO-A and *h*MAO-B inhibition and selectivity of the synthesised compounds (**1a**−1y).

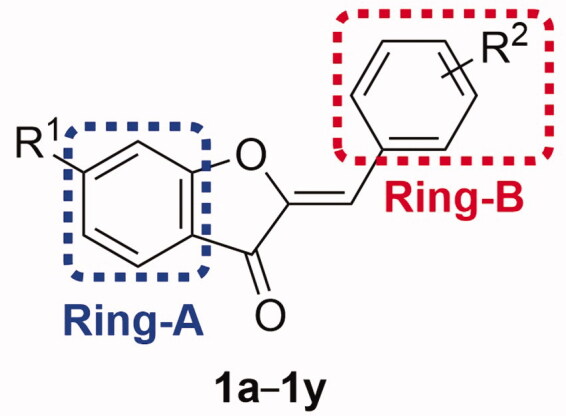							
Comp.	R^1^	R^2^	MAO-A		MAO-B		Selectivity index^c^
			% Inhibition^a^	IC_50_ (µM)^b^	% Inhibition^a^	IC_50_ (µM)^b^	
**1a**	6-Hydoxy	2′-Hydoxy	9.02	>10	46.77	>10	—
**1b**	6-Hydoxy	3′-Hydoxy	16.65	>10	13.56	>10	—
**1c**	6-Hydoxy	2′,3′-Dihydoxy	46.20	>10	**58.92**	**2.996**	>3.34
**1d**	6-Hydoxy	2′,4′-Dihydoxy	44.15	>10	48.34	>10	—
**1e**	6-Hydoxy	3′,4′-Dihydoxy	**64.89**	4.657	**61.79**	2.411	1.93
**1f**	6-Hydoxy	2′,5′-Dihydoxy	**53.31**	**7.774**	**61.39**	**3.522**	**2.21**
**1g**	6-Hydoxy	3′,5′-Dihydoxy	35.67	>10	12.77	>10	—
**1h**	6-Hydoxy	3′,4′,5′-Trihydoxy	49.48	>10	47.50	>10	—
**1i**	6-Hydoxy	2′-Methoxy	5.49	>100	**75.46**	**1.843**	>54
**1j**	6-Hydoxy	3′-Methoxy	7.43	>100	**73.17**	**2.414**	>41
**1k**	6-Hydoxy	4′-Methoxy	**52.98**	**6.978**	**94.59**	**0.365**	**>19**
**1l**	6-Hydoxy	2′,3′-Dimethoxy	−2.21	>100	**70.82**	**3.522**	>28
**1m**	6-Hydoxy	2′,5′-Dimethoxy	−0.29	>10	46.3	>10	—
**1n**	6-Hydoxy	3′,4′-Dimethoxy	4.52	>10	42.57	>10	—
**1o**	6-Hydoxy	3′,5′-Dimethoxy	0.37	>10	41.21	>10	—
**1p**	6-Hydoxy	2′,3′,4′-Trimethoxy	−1.53	>100	**96.70**	**0.171**	**>583**
**1q**	6-Hydoxy	4′-Methoxymethoxy	24.48	>100	**89.02**	**0.593**	**>168**
**1r**	6-Methoxy	2′-Hydoxy	2.29	>10	22.46	>10	—
**1s**	6-Methoxy	3′-Hydoxy	7.73	>10	48.85	>10	—
**1t**	6-Methoxy	4′-Hydoxy	8.54	>100	**89.6**	**1.333**	>75
**1u**	6-Methoxy	2′,3′-Dihydoxy	**52.83**	**7.041**	**65.04**	**2.414**	**>2.9**
**1v**	6-Methoxy	2′,4′-Dihydoxy	55.71	**8.205**	**66.69**	**6.042**	**>1.35**
**1w**	6-Methoxy	3′,4′-Dihydoxy	60.83	**5.623**	**70.5**	**2.414**	**>2.32**
**1x**	6-Methoxy	2′-Methoxy	21.65	>100	**89.46**	**0.593**	**>168**
**1y**	6-Methoxy	3′-Methoxy	3.26	>100	**99.23**	**0.201**	**>496**
Hispidol^d^			**69.28**	**5.282**	**34.62**	**>10**	**<0.53**
Safinamide^d^			—	—	**99.23**	**0.112**	—
Clorgyline^d^			100	**0.005**	—	—	—

^a^Percent inhibition of enzyme activity at a single dose concentration of 10 µM.

^b^IC_50_ values (µM) exhibited by the synthesised compounds.

^c^Selectivity index for inhibition of MAO-B was calculated by dividing IC_50_ values for inhibition of MAO-A over IC_50_ values for inhibition of MAO-B.

^d^Positive controls: hispidol: starting lead compound; safinamide: reversible MAO-B inhibitor; clorgyline: irreversible MAO-A inhibitor.

##### 2.3.1.1. Structure–activity relationship of *h*MAO-A inhibitory activity

The structure of hispidol involves a hydroxy substituent on ring A at C-6-position and mono- hydroxy substituent on ring B at *para*-position. These molecular features enabled hispidol to trigger micromolar MAO-A inhibitory activity in our assay system (IC_50_ was close to 5.28 µM, Table 1). Structure–activity analysis for C-6-hydroxy compounds with mono-hydroxylation pattern (*versus* hispidol) suggested an important role for ring-B *para*-position or a deleterious effect of ring-B *ortho*- or *meta*-positions mono-hydroxy substitution pattern. Thus, compounds **1a** and **1b** were of very low MAO-A inhibitory activity. Combining two-hydroxy substituents at ring-B *para*- and *ortho*-positions (compound **1d**) decreased MAO-A inhibitory activity relative to hispidol while at both ring-B *para*- and *meta*-positions maintained almost same level of MAO-A inhibitory activity (compound **1e**). Interestingly, vicinal *ortho*- and *meta*-dihydroxylation pattern at 2′,3′-positions should low activity (compound **1c**) while *ortho*- and *meta*-dihydroxylation pattern at 2′,5′-positions was more capable of inhibiting MAO-A activity but with lower potency relative to hispidol (compound **1f**). However, two hydroxy moieties at both *meta*-positions and ring-B as well as three hydroxy moieties at *para*- and both *meta*-positions elicited low MAO-A inhibitory activities (compounds **1g** and **1h**). Based on activities of ring-B positional scanning for hydroxy moieties in C-6 hydroxy compounds **1a**–**1h**, it might be deduced that ring-B *para*-position is the most important for eliciting MAO-A inhibition. Replacing the polar hydrogen bond donor and acceptor hydroxy moieties of ring-B with the less polar hydrogen bond donor only methoxy moieties (compounds **1i**–**1p**) reinforced the assumption of the importance of *para*-position. Thus, only compound **1k** having monomethoxy substituent at *para*-position of ring-B showed potential MAO-A inhibitory activity. As a methoxy substituent is more steric than hydroxy substituent and is also unable to accept hydrogen indicates some steric tolerance of this position as well as negates establishment of a donor-hydrogen bond at this position. However, such steric tolerance might be limited as compound **1k** showed relatively lower activity than hispidol. The finding that compound **1q** having the protruding methoxymethoxy substituent at *para*-position of ring-B showed limited MAO-A inhibitory activity provides further supported to this found steric tolerance. It was notable to find out that C-6 methoxy-substituted ring-A compounds **1r**–**1y** show MAO-A inhibitory activity profile distinct from compounds **1a**–**1q** having C-6 hydroxy-substituted ring-A. While the monomethoxy-substituted ring-B derivatives (**1x** and **1 y**) possessed very low to almost no MAO-A inhibitory activity similar to the trend found for C-6 hydroxy-substituted ring-A compounds, the activity trend was changed for monohydroxy-substituted ring-B derivatives (**1r**–**1t**) as no MAO-A inhibitory activity was triggered by *para*-substituted ring-B. In addition, the vicinal *ortho*- and *meta*-dihydroxylation pattern at 2′,3′-positions as well as *para*- and *ortho*-positions in C-6 hydroxy-substituted ring-A compounds (**1c** and **1d**, respectively) which were of low activity were found to trigger potential MAO-A inhibitory activity in C-6 methoxy-substituted ring-A compounds (**1 u** and **1v**, respectively). This activity trend change could reflect different binding modes for C-6 methoxy-substituted ring-A compounds.

##### 2.3.1.2. Structure–activity relationship of *h*MAO-B inhibitory activity

As human MAO-B isoform is different from human MAO-A, different structure-activity relationship trend might be identified which might assist in achieving selective inhibitors better than the poorly selective hispidol. Amongst compounds having ring-A C-6-hydroxy substitution pattern, *ortho*-monohydroxy substituted ring-B (compound **1a**) showed some increased MAO-B inhibitory activity relative to hispidol but the activity still under 50% inhibition. However, *meta*-monohydroxy substituted ring-B (compound **1b**) showed very weak MAO-B inhibition which was less than hispidol. In general, combining two-hydroxy substituents at ring-B resulted in slight to significant increase in MAO-B inhibitory activity (compounds **1c**–**1f**) except for combining two *meta*-hydroxy substituents on ring-B as well as three hydroxy moieties at both two *meta*-and *para*-positions (compounds **1g** and **1h**) which showed marked decreased activity. In fact, vicinal *ortho*- and *meta*-dihydroxylation pattern at 2′,3′-positions, as well as *ortho*- and *meta*-dihydroxylation pattern at 2′,5′-positions or *para*- and *meta*-dihydroxylation of ring-B afforded potential MAO-B inhibitors eliciting low micro molar IC_50_ values. It might be concluded that steric requirements are tolerated for dihydroxylation of ring-B at these positions. Replacing the polar hydrogen bond donor and acceptor hydroxy moieties of ring-B with the less polar hydrogen bond donor only methoxy moieties (compounds **1i**–**1p**) afforded an interesting structure-activity relationship outcome. There were significant increases in MAO-B inhibitory activities of monomethoxylated patterns of ring-B not only for *para*-methoxy substituent on ring-B (compound **1k**), which exhibited a submicromolar potency, but also *ortho*- or *meta*-methoxy substitution patterns at ring-B afforded potential MAO-B inhibitors eliciting low micro molar IC_50_ values (compounds **1i** and **1j**). This suggested not only a tolerance of steric requirements of the more demanding methoxy groups, but also negated establishment of hydrogen bond acceptor interactions and, further, might indicate hydrophobic interactions. Except for compound **1 l** having vicinal *ortho*- and *meta*-dimethoxy substituents at 2′,3′-positions, substitution patterns combining two methoxy substituents at ring-B (compounds **1 m**–**1o**) resulted in lowering MAO-A inhibitory activity under 50% inhibition. Such results suggested that steric demand of vicinal *ortho*- and *meta*-dimethoxy substituents at 2′,3′-positions of ring-B, but not other patterns, might be well-tolerated. Combining such vicinal *ortho*- and *meta*-dimethoxy substituents with a third methoxy group at *para*-position of ring-B, which was revealed to afford submicromolar potent compound **1k**, resulted in a more potent MAO-B inhibitor **1p** eliciting submicromolar activity comparable to safinamide. Such outcome suggested steric tolerance or favourable hydrophobic interactions of such trimethoxylated pattern of compound **1p**. The finding that compound **1q** having the protruding methoxymethoxy moiety at *para*-position of ring-B elicited potent submicromolar activity reinforces that conceived conclusion that MAO-B inhibitory activity might tolerate steric demanding substituents at this position. Next, structure–activity relationship for compounds having C-6-methoxy substitution pattern were analysed. For compounds, **1r**–**1t** bearing monohydroxy substitution pattern showed that only compound **1t** possessing this moiety at *para*-position elicited high inhibition and displayed low micromolar potency. Combining the two *ortho*- and *meta*-dihydroxy substituents the vicinal 2′,3′-positions as well as the *meta*- and *para*-positions in C-6-methoxy derivatives **1u** and **1w,** respectively, showed more or less similar activity to analogous derivatives in C-6-hydroxy derivatives **1c** and **1e**, respectively. However, the *ortho*- and *para*-dihydroxy groups at ring-B in C-6-methoxy derivative **1v** showed enhancement of activity relative to analogous derivative **1d** in C-6-hydroxy derivatives. The enhancement of activity was more remarkable for ring-B monomethoxylated derivatives **1x** and **1y** bearing *ortho*- or *meta*-substitution pattern, respectively, which elicited potent submicromolar activities. Remarkably, potency of compound **1y** possessing *meta*-methoxylation patterns of ring-B was comparable to safinamide.

##### 2.3.1.3. Selectivity of MAO-B inhibition

Analysis of activity data of hispidol and the synthesised 25 hispidol analogues against MAO-A and MAO-B isoforms revealed interesting outcome. Similar to recently reported benzo[*b*]tiophen-3-ol derivative that revealed dependence of the triggered MAO inhibitor activity on position and chemical nature of the substituent^60^, it became evident from our results that amongst ring-A C-6-hydroxy analogues **1a**–**1q**, there might be steric limitations for substituents at *ortho*- and *meta*- positions of ring-B. This was reflected in lowered MAO-A inhibitory activities of compounds having the sterically more demanding methoxy groups more than those having the less bulkier hydroxyl groups (e.g. compounds **1i** and **1j** *versus* compounds **1a** and **1b**). In contrast, MAO-B inhibition activity tolerated substituents at these positions and even derivatives incorporating the bulkier methoxy moieties at these positions elicited potential MAO-B inhibition. It was found that both potential MAO-A and MAO-B inhibition activities tolerated and were associated with substituents at *para*-position of ring-B (e.g. compounds **1k** and hispidol). Accordingly, derivatives having such substitution pattern, although were potential MAO inhibitors, showed low selectivity values. However, it was found that steric tolerance of MAO-A isoform is limited relative to tolerance of MAO-B. Accordingly, incorporation the more steric protruding methoxymethoxy at *para*-position of ring-B afforded potential MAO-B inhibitor **1q** showing high selectivity (Table 1, selectivity index > 168). Another strategy was revealed to afford selective MAO-B inhibition based on combining more than one substituent at ring-B to exceed the steric requirements of MAO-A but still tolerated by MAO-B. This strategy might be indicated by the highly selective potent MAO-B inhibition, yet almost no MAO-A inhibition, by compound **1p** (Table 1). This derivative combines *para*-methoxy moiety at ring-B; which was found to trigger both MAO-B and MAO-A inhibitory activities, with vicinal *ortho*- and *meta*-methoxy moieties at ring-B; which were found to abolish MAO-A inhibitory activity. Switching to ring-A C-6-methoxy analogues **1r**–**1y**, it was found again that MAO-B isoform, in contrast to MAO-A isoform, tolerated substituents at *ortho*- and *meta*-positions of ring-B. In addition, sterically more demanding and less polar methoxy moieties were efficient in triggering submicromolar MAO-B IC_50_ values while showing very low MAO-A inhibition. Consequently, compounds **1x** and **1y** were identified as highly selective potent MAO-B inhibitors. Collectively, four compounds; **1p**, **1q**, **1x,** and **1y**, were found to trigger highly selective potent MAO-B inhibition.

#### 2.3.2. Evaluation of absence of cells’ viability disruption

**Figure 2.**
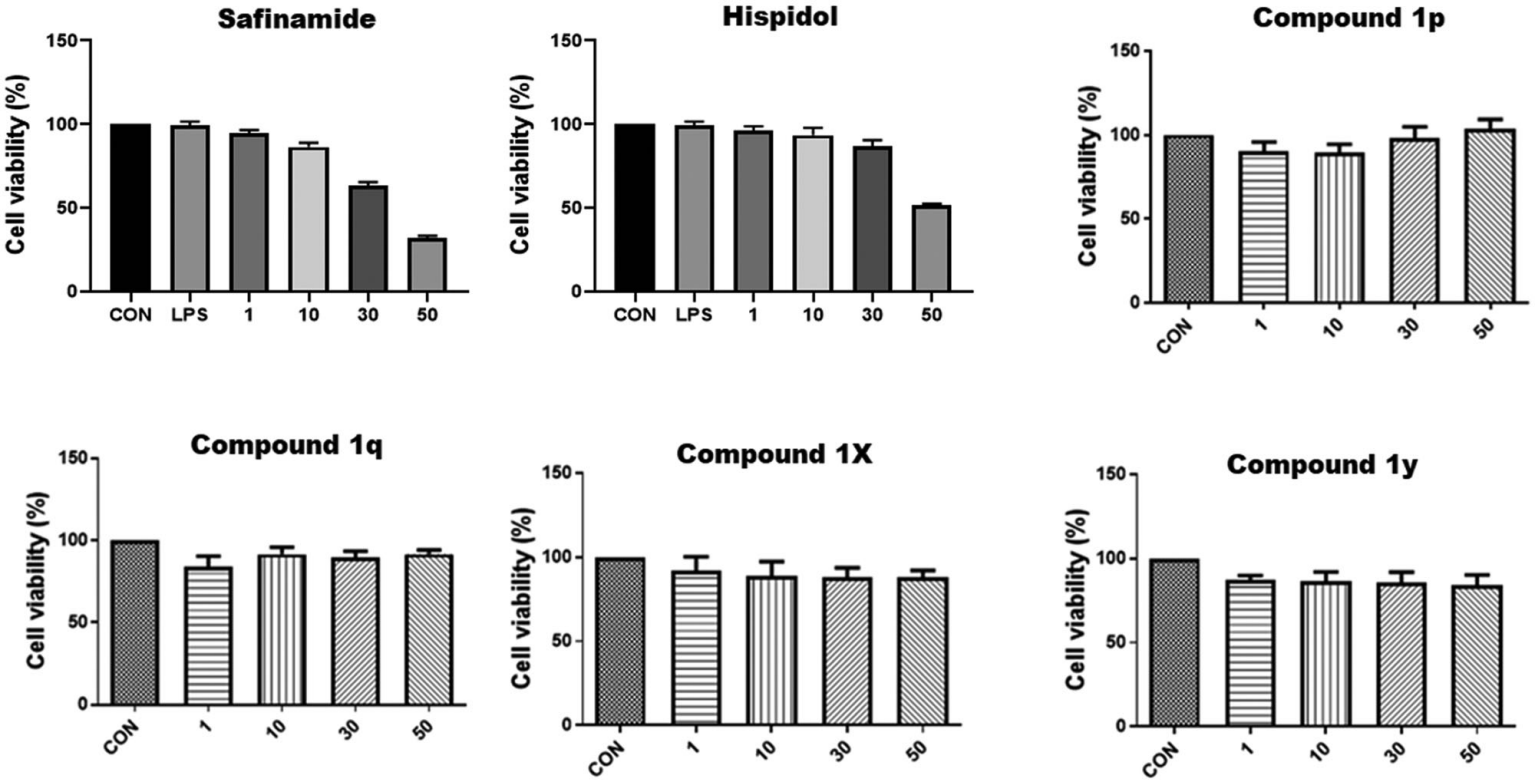
Dose-dependent viabilities % upon treatment with the evaluated candidate hispidol-analogues as well as safinamide and hispidol.

Promising antineurodegenrative candidate molecules should not impair viability of CNS cells. Microglia are critical cells within CNS involved in microglia–neuron communications and are responsible for maintenance of brain integrity and health^61^^,^^62^. However, under neuroinflammatory conditions, aberrant activation of microglia contributes to neurodegeneration and disease progression^63^. In this regard, suppression of the abnormal activation while maintenance of viability of microglia is important considerations. To assess this later crucial requirement, the identified potent MAO-B inhibitor compounds that showed selective MAO-B inhibition by 168-fold or more were evaluated for their impact on viability of BV2 cells; a valid *in vitro* model for microglia^64^. The viability assay was carried out using the water-soluble tetrazolium reagent WST-1 which eliminates the formazan dissolution step requested in case of MTT^65^ employing multiple doses of 1, 10, 30, and 50 µM. On the opposite to revealed cytotoxic effects for safinamide and hispidol at high doses (Figure 2), the results indicated that none of the tested concentrations for all of the employed four compounds **1p**, **1q**, **1x,** and **1y** triggered significant impairment of BV2 cells’ viability. In lieu of this, it might be inferred that these compounds showing effective inhibition of MAO-B activity and triggering no impairment of microglia viability are good candidates might be advanced for further evaluation towards the development of multifunctional molecules for neurodegenerative diseases such as PD.

#### 2.3.3. Investigation of anti-neuroinflammatory activity

**Figure 3.**
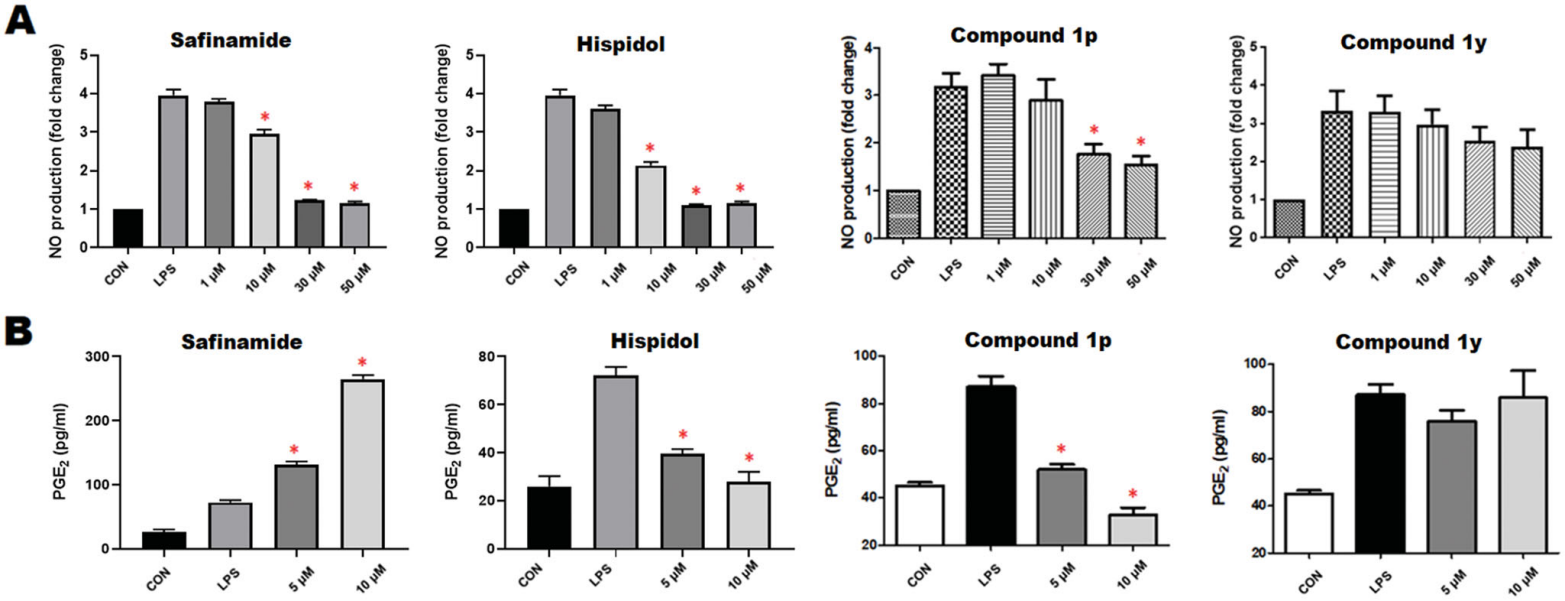
*In vitro* anti-neuroinflammatory activity in BV2 Cells: A) dose-dependent effects of compounds **1p** and **1y** as well as safinamide and hispidol against LPS-induced production of nitric oxide; B) Dose-dependent effects of compounds **1p** and **1y** as well as safinamide and hispidol against LPS-induced production of PGE_2_.

Neuroinflammation is a crucial component in neurodegenerative diseases. Chronic activation of microglia, which are the brain-resident form of macrophages, results in sustained production of several proinflammatory mediators including glial NO, PGE_2_, and others molecules that are implicated in multiple neurodegenerative diseases^66–68^. Considering the that LPS-induced production of NO and PGE_2_ in RAW 264.7 macrophages was found to be inhibited by this class of compounds^49^, the ability of these compounds to inhibit the production of proinflammatory mediators by CNS microglia was checked. In addition to safinamide and hispidol, which were used as controls, both of compounds **1p** and **1y** which emerged as potent (IC_50_ = 0.171 and 0.201 µM, respectively), highly selective MAO-B inhibitors (selectivity indices were >583 and >496 respectively) and, in addition, have no detrimental effects of cells viability were employed for these evaluations. In this regard, LPS-treated BV2 cells were incubated with or without different concentrations of each compound. As shown in Figure 3(A), compounds **1p**, safinamide, and hispidol but not compound **1y** triggering significant inhibition of NO production by BV2 cells. Interestingly, safinamide showed undesirable dose-dependent increase in PGE_2_ production at tested concentrations. Meanwhile, compound **1p** and hispidol effectively suppressed the induced-PGE_2_ production by BV2 cells at low concentrations (Figure 3(B)). Based on collective results of antineuroinflammatory activity evaluation, in conjugation with above-conducted MAO inhibitions’ assays, compound **1p** might be claimed as a promising lead compound eliciting polypharmacological effects combining inhibition of neuroinflammation and MAO-B activity; both processes are crucial components of neurodegenerative diseases such as PD.

### 2.4. *In vitro* MAO-B inhibition kinetic studies and evaluation of reversibility

**Figure 4.**
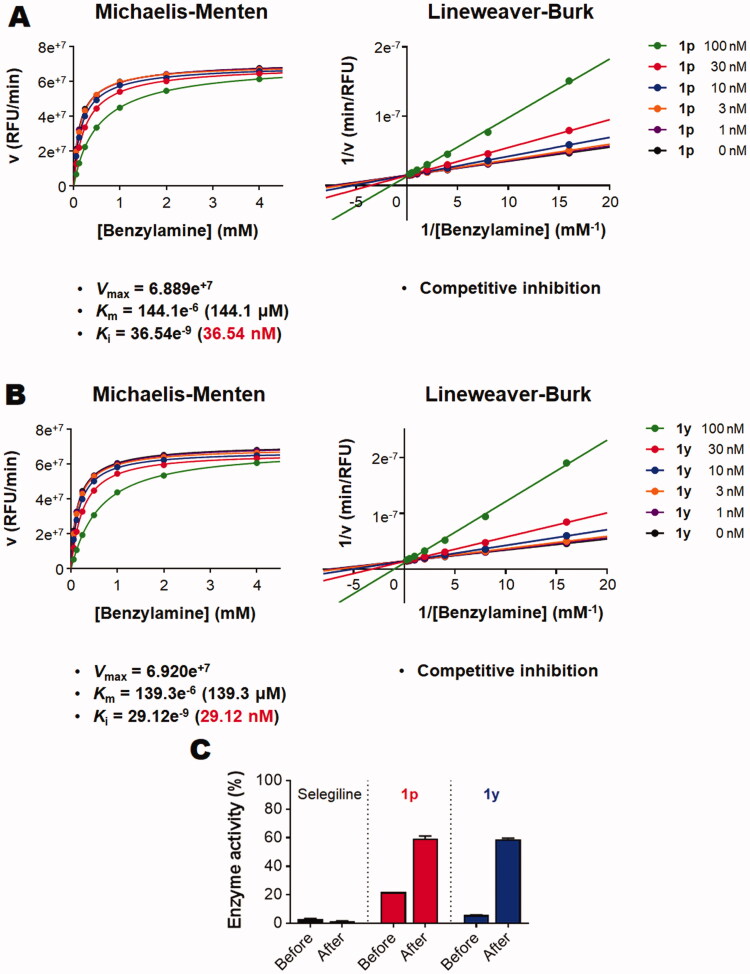
*In vitro* kinetic study and reversibility testing for inhibition of MAO-B by compounds **1p** and **1y**: (A) Kinetics of inhibition of MAO-B by different concentrations of compound **1p** employing different concentrations benzylamine as a substrate; (B) Kinetics of inhibition of MAO-B by different concentrations of compound **1y** employing different concentrations benzylamine as a substrate; (C) Reversibility testing for inhibition of MAO-B by compounds **1p** and **1y**.

To get insights into MAO-B inhibition reaction by compounds **1p** and **1y**, a kinetic study and reversibility testing were conducted. As illustrated in Figure 4(A,B), Michaelis–Menten graphs were constructed using different concentrations of compounds **1p** and **1y** employing different concentrations benzylamine as a substrate. Calculation of the reaction kinetics showed that compounds **1p** and **1y** are potent MAO-B inhibitors showing approximately similar V_max_, K_m_, and K_i_ values (6.89e^+7^ *versus* 6.92e^+7^, 144.1 *versus* 139.3 µM, and 36.5 *versus* 29.1 nM for V_max_, K_m_, and K_i_ of compounds **1p** *versus* **1y,** respectively). Establishing Lineweaver–Burk plots indicated that both compounds **1p** and **1y** are competitive inhibitors. Reversibility testing (Figure 4(C)) showed that washing restored the enzymatic activity in case of both of compounds **1p** and **1y** but not for selegiline (an irreversible MAO-B inhibitor) proving that both compounds are reversible MAO-B inhibitors.

### 2.5. *In-silico* simulation study

**Figure 5.**
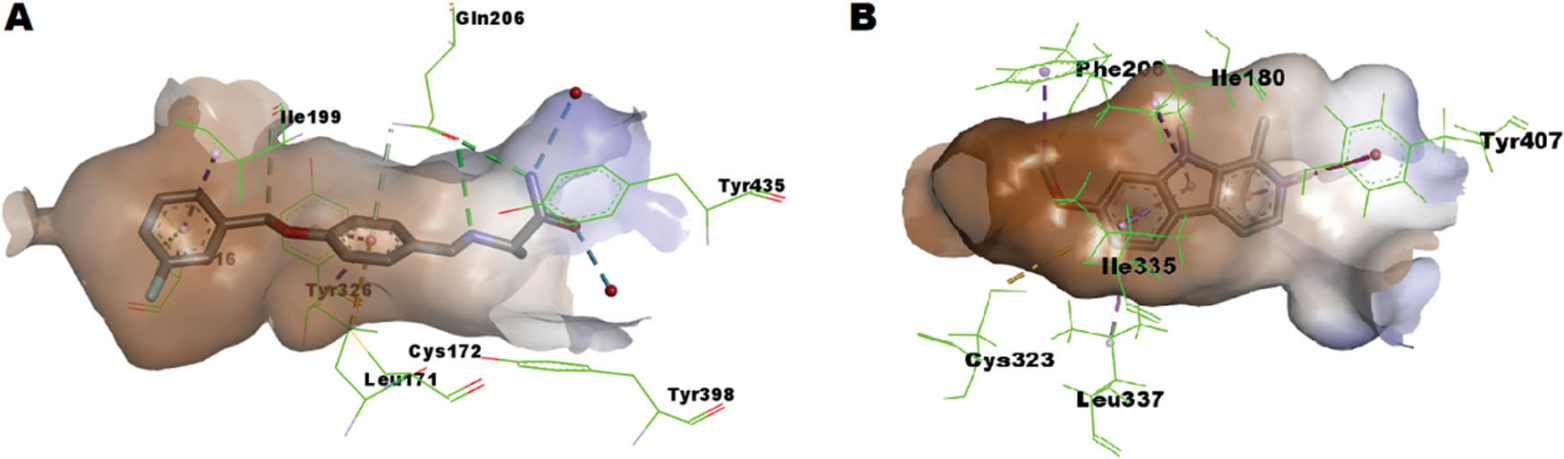
(A) Interactions network of safinamide within MAO-B pocket as found in the co-crystallised complex (PDB: 2V5Z); (B) Interactions network of harmine within MAO-A pocket as found in the co-crystallised complex (PDB: 2Z5X).

To get insights into the molecular interactions of the discovered compounds potentially active selective compounds, *in-silico* calculation of the possible binding modes of compounds **1p** and **1y** with MAO-B and MAO-A was conducted. The substrate-binding site of MAO-A is around 550 Å^3^ while MAO-B has a 420 Å^3^ substrate-binding cavity connected to a 290 Å^3^ hydrophobic entrance cavity. Amino acid residues Ile199 and Tyr326 of MAO-B are gating residues that can open to connect the two site into a single elongate cavity as in safinamide-MAO-B complex (Figure 5(A), PDB code: 2V5Z). In case of MAO-A, Ile335, and Phe208 are the gating residues which correspond to Tyr326 and Ile199 of MAO-B (Figure 5(B), harmine-MAO-A complex, PDB code: 2Z5X). The differences in size, shape and gating amino acid residues of substrate/inhibitor binding pocket might be the basis of selectivity of some inhibitors^69–71^.

**Figure 6.**
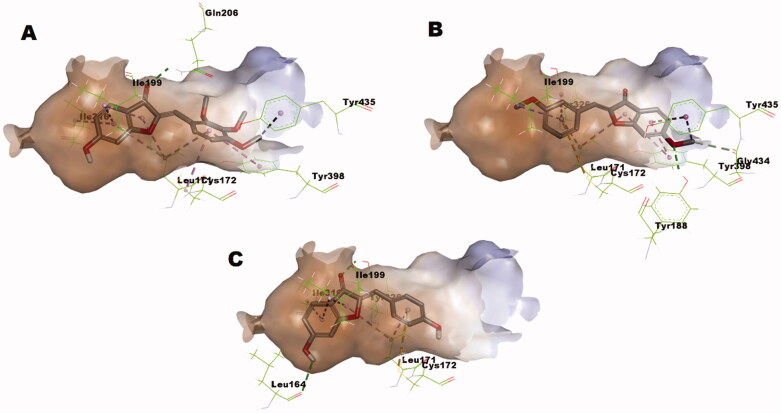
(A) Predicted binding mode of compound **1p** within MAO-B pocket (PDB: 2V5Z); (B) Predicted binding mode of compound **1y** within MAO-B pocket (PDB: 2V5Z); (C) Predicted binding mode of hispidol within MAO-B pocket (PDB: 2V5Z).

As illustrated in Figure 6(A), *in-silico* calculations predicted that compound **1p** well-fits in the substrate pocket of MAO-B showing an orientation that positions the 2,3,4-trimethoxybenzylidene moiety towards the aromatic cage formed of Tyr398 and Tyr435 in front of the flavin, while the coumaranone moiety extends into the remainder elongated hydrophobic pocket. Meanwhile compound **1y** well-fits in the substrate pocket of MAO-B in flipped orientation where the coumaranone moiety was directed towards the aromatic cage formed of Tyr398 and Tyr435 in front of the flavin, while the 3-methoxybenzylidene moiety extended into the remainder elongated hydrophobic pocket (Figure 6(B)). Both compounds established important interactions with aromatic cage amino acids; Tyr398 and Tyr435 forming *π*–*σ* interaction through the 4′-methoxy group and *π*–*π* interaction through the benzylidene ring of compound **1p**, and through *π*–*σ* interaction with the 6-methoxy group and *π*–*π* interactions through rings A and C of compound **1y**. In addition, they formed favourable interaction with the gating amino acid residue Ile199 through *π*-alkyl interaction with rings A and C of compound **1p** and the ring C of compound **1y**. However, the docked hispidol did not show the important interactions with aromatic cage amino acids; Tyr398 and Tyr435 but showed interactions with the gating amino acid residue Ile199 (Figure 6(C)), which might explain its poor MAO-A inhibitory activity. The most potent compound **1p** showed also further interactions including hydrogen bonding through the carbonyl moiety with the other gating amino acid residue Tyr326. Such interaction was not noticed for compound **1y**. In addition, the calculated scores for docked poses showed slight favourable energy difference for compound **1p** over compound **1y** (−7.84287 *versus* − 7.79966, respectively). The predicted interaction network suggests important contribution of the methoxy groups in conjugation of rings A − C to the overall calculated binding mode.

**Figure 7.**
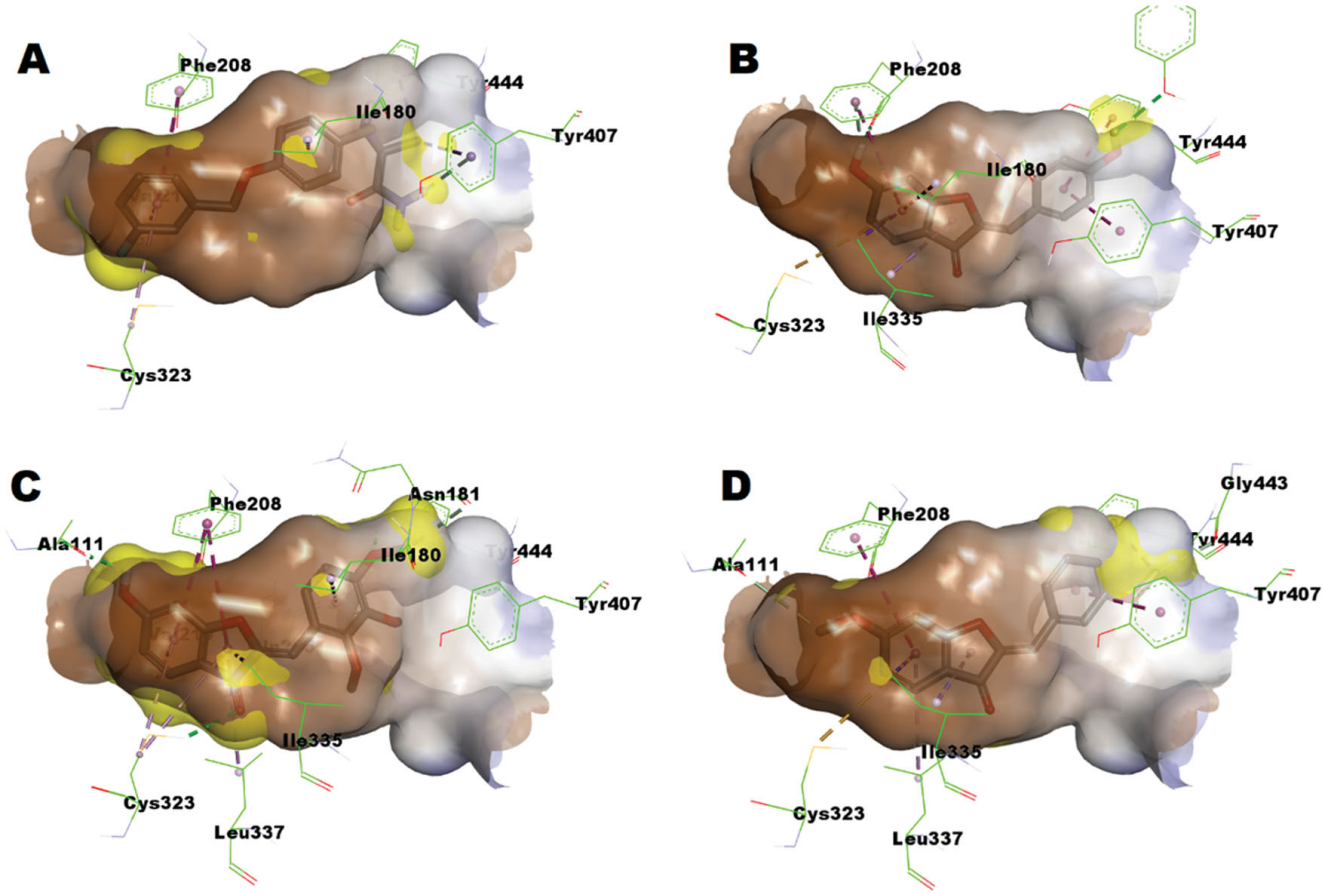
(A) Predicted binding mode of safinamide within MAO-A pocket (PDB: 2Z5X): Yellow surface represents the solvent-accessible surface of safinamide; (B) Predicted binding mode of hispidol within MAO-A pocket (PDB: 2Z5X): Yellow surface represents the solvent-accessible surface of hispidol; (C) Predicted binding mode of compound **1p** within MAO-A pocket (PDB: 2Z5X): Yellow surface represents the solvent-accessible surface of compounds **1p**; (D) Predicted binding mode of compound **1y** within MAO-A pocket (PDB: 2Z5X): Yellow surface represents the solvent-accessible surface of compounds **1y**.

As illustrated in Figure 7(A), attempts to dock safinamide; a MAO-B selective drug, into MAO-A revealed that MAO-A pocket size is smaller to accommodate it and produced a docked pose showing bumping of safinamide into the boundary of MAO-A pocket. This might provide a basis for the known selectivity of safinamide for MAO-B over MAO-A. Meanwhile, hispidol upon docking showed a docking pose that is free from bumping interactions and, furthermore, established important interactions with the aromatic cage residues (Figure 7(B)), which explains it activity as potential MAO-A rather than MAO-B inhibitor. Similar to safinamide, *in silico* calculations showed that the size of compound **1p** is larger than the substrate pocket size of MAO-A (Figure 7(C)). The calculation showed that the best pose of compound **1p** within MAO-A adopted in similar orientation to that observed in case of MAO-B. Nevertheless, the smaller substrate pocket of MAO-A resulted in unfavourable bumping into the pocket’s boundary of MAO-A which was absent in case of MAO-B. This size limitation might explain the high selectivity of compounds **1p**. On contrast, the size limit was less influential in case of compound **1y** which possessed smaller size and, thus, showed better fitting (Figure 7(D)). However, only one *π*–*σ* interaction with the 3′-methoxy group and one *π*–*π* interaction through the benzylidene ring of compound **1y** were predicted with the aromatic cage which is much less than the three π–*π* interactions through rings A and C of compound **1y** and through *π*–*σ* interaction with the 6-methoxy group. In addition, the larger distance of 5.20 Å for the interaction with gating Phe208 of MAO-A compared with a distance of 4.83 Å for interaction with the corresponding gating Tyr326 of MAO-B might be an additional factor contributing to its decreased propensity to inhibit MAO-A.

## 3. Conclusion

Neurological disorders including neurodegenerative and neuropsychiatric diseases are diverse complex diseases. Nevertheless, they share commonalities including anomalous monoaminergic pathways and neuroinflammation. To break the vicious circle of the disease, it might address multitargeted therapy instead of monotargeted therapy. In this regard, polypharmacological molecules might provide promising performance. Considering these points, this work was conducted in search for multifunctional molecules inhibiting MAOs and alleviating neuroinflammation. Hispidol, which is an aurone natural product reported to inhibit MAO-A with low selectivity was selected as a starting point to prepare a library of 25 analogues bearing different substitution patterns at ring-B. While previously reported aurones bearing only hydroxy/methoxy functions showed low selectivity for MAO inhibition, several members among the evaluated library members were identified as highly selective MAO-B inhibitors. As promising agents for treatment of neurodegenerative diseases should not impair cells viability, the most promising molecules were evaluated for their impact on viability of CNS microglia which presented them as safe compounds. Evaluation of the most promising compounds for anti-neuroinflammatory effects presented compound **1p** as a promising compound selectively inhibit MAO-B and downregulating neuroinflammation. Study of kinetics of the enzymatic inhibition reaction coupled with reversibility testing proved that compounds **1p** and **1y** are competitive reversible MAO-B inhibitors. Collectively, this report reveals compound **1p** as a promising multifunctional lead molecule for further development into possible anti-neurodegenerative therapeutics.

## 4. Experimental

### 4.1. Chemistry

*General:* All solvents and reagents have been purchased from commercial suppliers and used without any further purification. NMR spectra were acquired on Bruker Avance 400 spectrometer (400 MHz) or JEOL JNM-ECZ500R spectrometer (500 MHz). ^1^H NMR spectra were referenced to tetramethylsilane (*δ* = 0.00 ppm) as an internal standard. High-resolution mass spectra (HRMS) were recorded on Jeol AccuTOF (JMS-T100TD) equipped with a DART (direct analysis in real time) ion source from ionsense, Tokyo, Japan in the positive modes. TLC was carried out using glass sheets pre-coated with silica gel 60F_254_ purchased by Merk and spots were visualised under UV lamp or using staining solutions, such as *p*-anisaldehyde solution, ninhydrin solution. 6-Methoxy-3-coumaranone and compounds **1a**, **1b**, **1d**, **1e**, **1f**, **1h–1t**, and **v–1y** were in agreement with reported literature (Supplementary materials)^43^^,^^49^^,^^53–58^.

#### 4.1.1. General method for acid-catalysed cross-aldol condensation to prepare hispidol analogues

HCl was added dropwise to a solution of the appropriate 3-coumaranone derivative in methanol or ethanol. After complete dissolution, the appropriate benzaldehyde derivative was added dropwise. The mixture was heated at 60 °C for the specified time. After completion of the reaction (TLC), the reaction mixture was diluted with water, filtered and the collected precipitate was collected by filtration and dried under reduced vacuum before purification of the obtained crude products using column chromatography.

#### 4.1.2. General method for base-catalysed cross-aldol condensation to prepare hispidol analogues

KOH was added dropwise to a solution of the appropriate 3-coumaranone derivative in methanol or ethanol. After complete dissolution, the appropriate benzaldehyde derivative was added dropwise. The mixture was heated at 60 °C for the specified time. After completion of the reaction (TLC), the reaction was quenched by water, evaporated under reduced pressure and the obtained residue was partitioned between water and ethyl acetate, extracted with ethyl acetate, dried over MgSO_4_, evaporated under reduced pressure, and purified using column chromatography.

##### 4.1.2.1. (Z)-2–(2,3-Dihydroxybenzylidene)-6-hydroxybenzofuran-3(2H)-one (1c)

Compound **1c** was obtained according to general procedure 4.1.2 using 6-hydroxy-3-coumaranone (300.6 mg, 2.0 mmol), methanol (30 ml), KOH (50%, 3 ml) and 2,3-dihydroxybenzaldehyde (276.2 mg, 2.0 mmol). Reaction time was 6 h and the crude product was purified by column chromatography (silica gel, EtOAc/*n*-hexane = 1:3) to afford the above titled compound **1c** (80.7 mg, 19% yield).

^1^H NMR (500 MHz, MeOD-d_4_): *δ* 7.67 (d, *J* = 1.4 Hz, 1H), 7.66 (d, *J* = 1.4 Hz, 1H), 7.60 (d, *J* = 1.1 Hz, 1H), 7.59 (d, *J* = 1.2 Hz, 1H), 7.33 (s, 1H), 6.80 (dd, *J* = 8.0, 1.7 Hz, 2H_'_), 6.74 (t, *J* = 8.0 Hz, 2H), 6.68 (s, 1H), 6.66 (d, *J* = 1.7 Hz, 1H); ^13 ^C NMR (125 MHz, MeOD-d_4_): *δ* 183.4, 168.7, 167.1, 147.5, 146.2, 145.1, 125.6, 122.0, 119.5, 119.2, 116.3, 113.5, 112.7, 106.9, 98.1; HRMS calcd for C_15_H_11_O_5_ [M + H]^+^ 271.0607, found 271.0601.

##### 4.1.2.2. (Z)-2–(3,5-Dihydroxybenzylidene)-6-hydroxybenzofuran-3(2H)-one (1g)

Compound **1 g** was obtained according to general procedure 4.1.1 using 6-hydroxy-3-coumaranone (144.8 mg, 1.0 mmol), ethanol (30 ml), HCl (12 N, 3 ml) and 3,5-dihydroxybenzaldehyde (133.3 mg, 1.0 mmol). Reaction time was 6 h and it was purified by column chromatography (silica gel, EtOAc/*n*-hexane = 1:2) to afford the above titled compound **1 g** (25.3 mg, 7% yield).

^1^H NMR (500 MHz, DMSO-d_6_): *δ* 11.2 (brs, 1H), 9.47 (s, 2H), 7.59 (d, *J* = 8.4 Hz, 1H), 6.78 (d, *J* = 2.0 Hz, 2H), 6.68 (m, 2H), 6.52 (s, 1H), 6.27 (t, *J* = 2.2 Hz, 1H_'_); ^13 ^C NMR (125 MHz, DMSO-d_6_): *δ* 182.0, 168.4, 167.1, 159.1, 147.7, 133.8, 126.6, 113.6, 113.3, 111.6, 109.8, 105.0, 98.9; HRMS calcd for C_15_H_11_O_5_ [M + H]^+^ 271.0607, found 271.0623.

##### 4.1.2.3. (Z)-2–(2,3-Dihydroxybenzylidene)-6-methoxybenzofuran-3(2H)-one (1u)

Compound **1u** was obtained according to general procedure 4.1.1 using 6-methoxy-3-coumaranone (328.9 mg, 2.0 mmol), ethanol (10 ml), HCl (12 N, 3 ml) and 2,3-dihydroxybenzaldehyde (277.7 mg, 2.0 mmol). Reaction time was 8 h and the crude product was purified by column chromatography (silica gel, EtOAc/*n*-hexane = 1:2) to afford the above titled compound **1u** (147.1 mg, 26% yield).

^1^H NMR (500 MHz, DMSO-d_6_): *δ* 7.64 (d, *J* = 8.6 Hz, 1H), 7.57 (dd, *J* = 8.0, 1.5 Hz, 1H), 7.14 (s, 1H), 7.11 (d, *J* = 2.2 Hz, 1H), 6.82–6.79 (m, 2H), 6.71 (t, *J* = 7.9 Hz, 1H), 3.88 (s, 3H), 3.12 (s, 1H), 2.49 (s, 1H); ^13 ^C NMR (125 MHz, DMSO-d_6_): *δ* 182.1, 168.3, 167.7, 147.2, 146.8, 146.1, 125.9, 121.8, 119.9, 119.8, 117.4, 114.6, 113.1, 106.5, 97.7, 40.9; HRMS calcd for C_16_H_13_O_5_ [M + H]^+^ 285.0763, found 285.0764.

### 4.2. Biological evaluations

#### 4.2.1. *In vitro* assay of human monoamine oxidases inhibition

The inhibitory activities of the compounds against *h*MAO-A and *h*MAO-B enzymes were evaluated referring to a previously described method (Supporting information)^49^.

#### 4.2.2. *In vitro* evaluation of cell viability

The impact of the compounds on the viability of cells was performed by the well-known WST-1 assay method as described in the Supporting information.

#### 4.2.3. *In vitro* evaluation of inhibition of LPS-induced BV2 microglial cells production of nitric oxide and PGE2

The abilities of compounds to inhibit the induced production of NO and PGE_2_ were assessed by well-known protocol as indicated in the Supporting information.

#### 4.2.4. *In vitro* MAO-B inhibition kinetic studies and evaluation of reversibility

Kinetics of the inhibition of MAO-B and testing of reversibility were conducted according to literature-reported protocols^59^ as indicated in the Supporting information.

### 4.3. Molecular modelling study

Molecular docking was conducted using MOE employing MAO-B crystal structure (PDB: 2V5Z) and MAO-A crystal structure (PDB: 2Z5X) similar to literature reported protocol^72^.
